# Histopathological effects of the fruit extract of *Citrullus colocynthis* on the integument of the female tick *Hyalomma dromedarii*

**DOI:** 10.1038/s41598-026-46500-2

**Published:** 2026-04-17

**Authors:** Ashraf Ahmed Montasser, Salma Nabil Ahmed Mohamed, Asmaa Ali Baioumy Ali

**Affiliations:** Zoology Department, Faculty of Science, Ain Shams University, Abbassia, Cairo, 11566 Egypt

**Keywords:** *Citrullus*, Extract, Histology, *Hyalomma*, Integument, Volatiles, Biological techniques, Diseases, Ecology, Ecology, Zoology

## Abstract

*Hyalomma dromedarii* is a hard tick species parasitizing domestic animals, particularly camels. Heavy infestation results in huge economic losses through severe blood loss and transmission of pathogens, in addition to crucial problems for camel production. Worldwide control of ticks is mainly based on acaricides, which have led to environmental pollution, resistance development, and an increase in the cost of control. To reduce the drawbacks of chemical acaricides, new tick control methods are therefore required, such as the application of natural plant extracts. *Citrullus colocynthis*, commonly known as bitter apple, is a desert plant found in Egypt. It has an economic importance due to its bioactive compounds with antidiabetic, antimicrobial, and potentially anticancer properties. In addition, it is used as a natural preservative, as it was historically applied to protect Egyptian manuscripts and leather from fungal damage. The goal of this work was to study the histopathological and ultrastructural changes of *H. dromedarii* integument after immersion in 100 mg/mL of *C. colocynthis* ethanolic extract. Volatile components of the extract were detected following the use of gas chromatography-mass spectrometry (GC–MS). Light, scanning, and transmission electron microscopy examinations provided evidence that *C. colocynthis* caused great damage to the integument. Increasing eroded areas with irregular folds and warts were observed by SEM. LM and TEM showed integumental layers separation, procuticle disorganization, subcuticular layer rupture and epidermal layer damage. GC–MS revealed volatile constituents, such as methyl linoleate, octadecadienoic, palmitic, and stearic acids. This is the first histological investigation that reported the integumentary damage caused by *C. colocynthis* in *H. dromedarii*. The present data suggest that the changes in all integument layers of the female tick *H. dromedarii* following treatment with *C. colocynthis* extract may facilitate the transport of toxic compounds into ticks’ internal systems, which can then affect other organs. As a result, *C. colocynthis* can be considered as a promising tick control agent.

## Introduction

Ticks are significant ectoparasites that spread disease through their saliva to both humans and animals^1^, consequently, there is a pressing need for their natural management^2^. *Hyalomma dromedarii* is one of a significant livestock ectoparasites^3^. In many parts of the Middle East, it is the biggest barrier to camel production^4^. According to Champour et al.^5^, it causes mortality, reduces weight and milk production and damages leather production, as well as reduces reproduction by lowering pregnancy rates and raising abortion rates^6^.

Because the role of *Hyalomma dromedarii* in the transmission of various infectious diseases, such as Crimean-Congo hemorrhagic fever virus^7^, Dhori virus^8^, theileriosis of cattle (*Theileria annulata*)^9^, and theileriosis of camels (*T. camelensis*)^10^, it has received more attention^11^. So, we should manage this parasite to reduce the spread of these tick-borne diseases.

The traditional method of tick control, which involves chemical acaricides usage, is currently inadequate and unsustainable^11^. This is because of the emergence of resistant ticks, environmental contamination, residues in livestock products, repeated applications, and the impossibility and/or high cost of creating new acaricide generations in the near future^12^. For these reasons, scientists are searching for alternatives to get around the drawbacks of synthetic acaricides^13^. Utilizing plant extracts is one of the alternate strategies^2,14^. This is due to its biodegradability, lack of environmental accumulation and contamination, rarity of environmental and non-targeted species toxicity, and decreased likelihood of resistance development^15,16^.

*Citrullus colocynthis* is one of economically and biochemically important plants from Cucurbitaceae family^17^. Its fruit contains a variety of bioactive substances including fatty acids, flavonoids, alkaloids, glycosides, and essential oils^18^. It has exceptional pharmacological qualities such as anti-inflammatory, anti-tumor, anti-microbial, anti-mycotic, and antioxidant activities^19–21^.

The various extracts of *C. colocynthis* demonstrated anthelminthic activity on *Haemonchus contortus*^22^, molluscidal activity against *Biomphalaria arebica*^23^, insecticidal effects against *Tribolium castaneum*^24^ and *Lipaphis erysimi*^25^, antileishmanial activity against *Leishmania*^26^, and acaricidal activity against various tick stages, including *H. analoticum*^27^, *Rhipicephalus* sp.^28^ and *H. dromedarii*^2,29^.

Tick integument histological and ultrastructural changes after treatment with plant extracts and their natural components were studied in *R. sanguineus*^30–32^, *Haemaphysalis longicornis*^16^, and *H. dromedarii*^33^. Therefore, the aim of this study is to examine the histological and ultrastructural effects of an ethanolic extract of *C. colothynthis* on *H. dromedarii* integument. Additionally, gas chromatography-mass spectrometry (GC–MS) was used to identify volatile components in order to investigate the mode of action of the plant extract on the tick integument.

## Methods

### Tick collection

*Hyalomma dromedarii* were collected from camels naturally infested with ticks at the Birqash camel market (30° 9′ 58.4″ N, 31° 2′ 13.2″ E), Giza Governorate, Egypt. Based on Hoogstraal and Kaiser^34^, ticks were identified, then grouped into non-, semi-, and engorged adults (males and females)^35^. Within 24 h of collection, engorged females were put in glass vials wrapped with gauze pieces. They were kept in an incubator at 28 ± 2 °C and 75–80% relative humidity until time of the experiments.

### Preparation of the extract

*Citrullus colocynthis* ripen dried fruits were bought from the market. Plant materials were identified by Dr. May Taha, assistant professor in Botany Department, Faulty of Science, Ain shams University, and some were kept at CAIA (Ain Shams University Herbarium). Dried fruits were cleaned to remove dust, and ground using a stainless-steel knife mill. The method of Twaij et al.^36^ was followed in the preparation of ethanolic extract. Plant powder (50 g) was added to 80% ethyl alcohol (250 mL), covered with aluminium foil and kept in dark condition for 72 h, then it was filtrated using Whatman filter paper (110 mm diameter opening). The filtrate was poured into glass Petri dishes and placed in the incubator at 50 °C to evaporate the alcohol. The dried extract was then gathered, weighed, placed in glass vials, and stored at 4 °C until use. To prepare a concentration of 100 mg/mL (LC_50_ according to Mahamed et al. 2), 1 g of the extract was dissolved in 10 mL of distilled water.

### Treatment

The adult immersion test (AIT) was conducted according to Drummond et al.^37^. Each engorged tick female was immersed in 10 mL of 100 mg/mL for 5 min (LC_50_ according to Mahamed et al.^2^). Then they were placed in sterile glass vials securely covered by gauze. Both untreated and treated specimens were kept inside incubator adjusted at 28 ± 2 °C temperature and 75–80% relative humidity.

### Morphological and histological studies

Untreated and treated females were dissected after 4 and 7 days of engorgement and treatment. Throughout the experiments, 63 spicemens were dissected. For the untreated and treated specimens at each period, three replicates each consisting of 3 females were maintained.

#### Light microscopy

Dissection of tick females occurred under a dissecting binocular microscope. Females were covered with 0.85% NaCl solution in a Petri dish filled with a mixture of charcoal and paraffin wax. After removing the dorsal integument, it was repeatedly cleaned with saline solution and fixed in Bouin’s fixative (aqueous-based solution consists of saturated picric acid, formaline and glacial acetic acid) for 24 h^38^. Then they dehydrated in ascending series of the ethyl alcohol before being placed for 24 h in methyl benzoate. Samples were placed in 2% celloidin in methyl benzoate solution for 24 h^39^, cleared in benzol, submerged in three paraplast changes at 56 °C, and finally embedded in the paraplast (Fisher Scientific Inc. USA).

Serial transverse sections were cut 3 µm in thick using computer microtome (YD-335-Huran Kaida Scientific Instrument Comp., China), and stained with either Mallory triple stain (MT)^40^ or hematoxylin–eosin stain (HE)^41^. Sections were photographed using a digital camera (Samsung ES95 HD) fixed on a microscope (Olympus, Japan made).

#### Electron microscopy

Females were dissected in cold phosphate buffer (pH 7.2). After removing integument, it was fixed for 2 h in 3% cold fresh glutaraldehyde. Phosphate buffer was used to wash the samples for 30 min., then the following procedures were done for scanning and transmission electron microscopy.

##### Scanning electron microscopy (SEM)

Integument was dehydrated in ascending series of ethanol. It subjected to critical point drying, attached to aluminum stubs, and coated with gold using a sputter-coating apparatus. Then it examined and photographed under a Quanta (FEG 250) scanning electron microscope (FEI Company, Hillsboro, Oregon, USA) at the Electron Microscope Unit, Desert Research Center, Cairo, Egypt.

##### Transmission electron microscopy (TEM)

Integument samples were postfixed in cold 1% osmic acid for 2 h and washed again in fresh buffer. They were dehydrated in ascending series of ethanol and embedded in an epoxy resin^39^.

Semithin sections (500 to 1000 nm) were cut using Leica Ultra-cut (UCT ultra-microtome) and stained with toluidine blue stain (TB)^42^. Then ultrathin sections (75–90 nm) were cut using a diamond knife and the same ultratome, mounted on copper grids (grid size 300 mesh × 83 µm pitch), and stained with uranyl acetate and lead citrate^43^. Integument samples were examined by JEOL (JEM-1400 TEM) transmission electron microscope at the Electron Microscope Unit, Faculty of Agriculture, Cairo University, Egypt.

### Gas chromatography-mass spectrometry (GC–MS) analysis

#### Diethyl ether extraction

Volatile components from *C. colocynthis* were extracted by the solvent extraction (SE) method^44^. Three grams of plant powder were extracted using diethyl ether (1:10, w/v) for three times (15 min each time) using ultrasonic. The solution was filtrated, and the solvent was removed using rotary evaporation under low pressure. Then the extract was diluted with 1 mL of anhydrous ethyl alcohol: *n*-hexane (1:1, v/v) then filtered through a membrane filter (0.22 μm). The subsequent filtrate (1 μL) was injected to GC–MS for analysis.

#### GC–MS analysis

The analysis for volatiles was performed by the GC–MS instrument (Thermo Electron Corporation, USA) equipped with a Finnigan Trace DSQ and an electron impact (EI) ion source. The analytes were separated on a DB-5MS capillary column (30 m × 0.25 mm × 0.25 μm; Agilent, USA) coated with phenyl arylene polymer. The oven temperature program was: 50 °C initially for 1 min, increased to 145 °C at °C/min, increased to 175 °C at 7 °C/min, increased to 195 °C at 5 °C/min, and then ramped to 250 °C at 3 °C/min; 250 °C was maintained for 10 min. High pure helium (99.999%) was the carrier gas set at a constant flow rate (1 mL/min). The injection port, ion source and transfer line temperatures were set at 250 °C. 70 eV of EI was adopted, and the mass scanning range was set from 50 to 650 amu in full scan. The injection was performed by split mode with a split ratio of 10:1. For all samples generated by different methods, the solvent delay time was set for 3 min. Xcalibur 2.0 workstation was used to process data. Most volatile components were identified by comparison of their retention times and obtained mass spectra of the analytes with those of authentic standards from the NIST libraries (2005) and with the mass spectra published previously^45–47^. Peak areas of all components were calculated by Xcalibur 2.0.

The protocol and procedures were approved by the Research Ethics Committee, Faculty of Science, Ain Shams University. Code: ASU-SCI/ZOOL/2024/7/1.

## Results

**Fig. 1:**
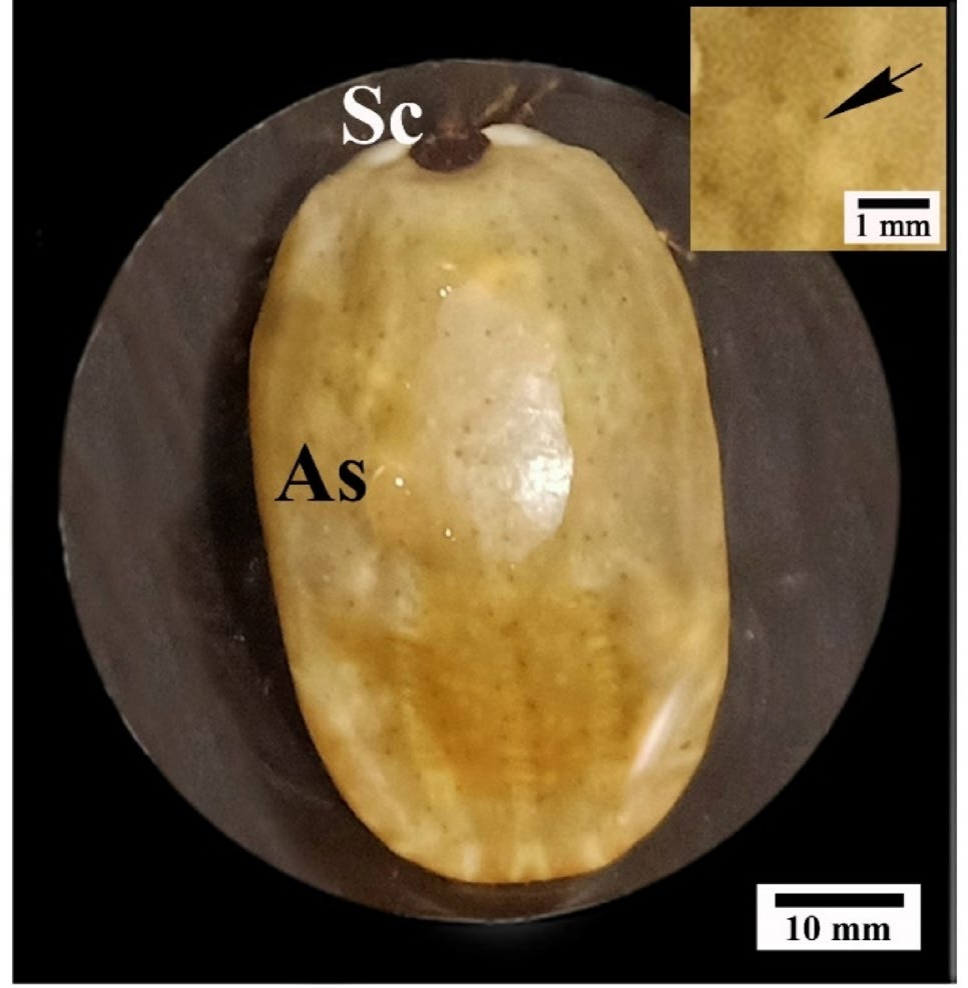
Photograph of dorsal view of untreated female *H. dromedarii* showing dorsal integument with anterior scutum (Sc) and posterior alloscutum (As) with many dermal gland openings (arrow in enlarged portion).

The female *H. dromedarii* has an oval body with a posterior alloscutum having festoones at the posterior border, as well as an antero-dorsal sclerite (scutum) (Fig. 1). On the dorsal surface, dermal gland apertures were dispersed (Fig. 1).

**Fig. 2:**
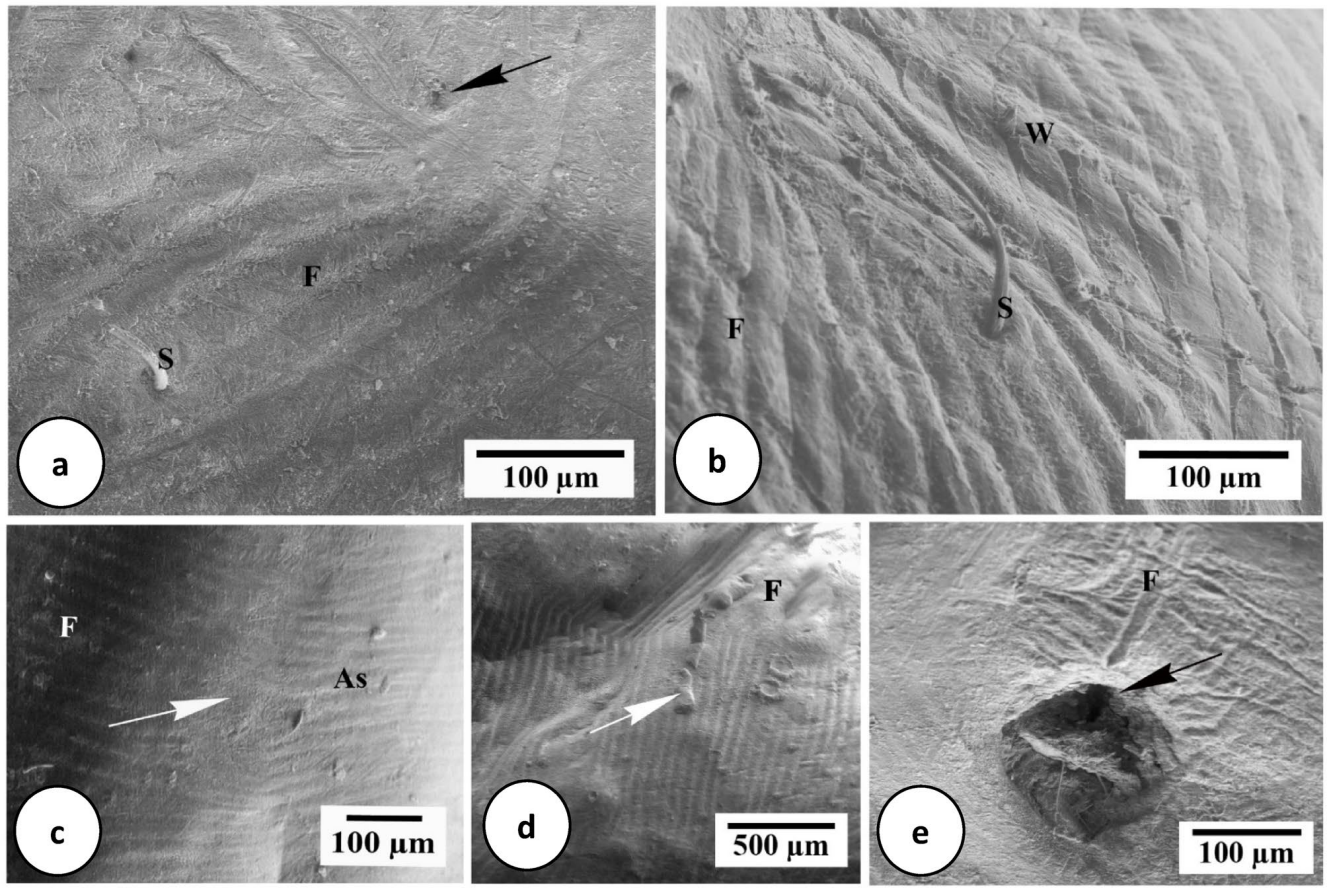
Scanning electron micrographs of *H. dromedarii* integument. (**a**) 4 days after feeding showing slight folds (F), setae (S) and openings of dermal glands (arrow). (**b**) 7 days after feeding showing numerous folds (F), setae (S) and outer wax layer (W). (**c**) 4 days after treatment showing alloscutum (As), folds (F) and integumental eroded area (arrow). (**d**) 7 days after treatment showing warts on the surface (arrows) with abnormal appearance of folds (F). (**e**) 4 days after treatment showing the blockage and deformation of dermal gland opening by secretions (arrow) and irregular folds (**F**).

Under scanning electron microscopical examination, dorsal integument of engorged female *H. dromedarii* is clearly expanded, with minor foldings and dispersed setae (Fig. 2a,b). Four and 7 days following feeding, there were no alterations in the alloscutum surface structure. Seven days after feeding, there was an increase in folds number (Fig. 2b). The wax substance on the external part of the integument (Fig. 2b) indicates that the dorsal integument has some apertures of dermal glands (Fig. 2a), which discharge their secretions to the cuticle surface.

Comparing treated and untreated female *H. dromedarii* integument at the aforementioned treatment stages revealed significant alterations. Four days following treatment, the alloscutum displayed damage and morphological deformation (Fig. 2c). The dorsal integument had several minor eroded areas (Fig. 2c). The integument showed increasing eroded areas with irregular folds and warts seven days after treatment (Fig. 2d). The secretion of the dermal gland blocked its opening (Fig. 2e).

**Fig. 3:**
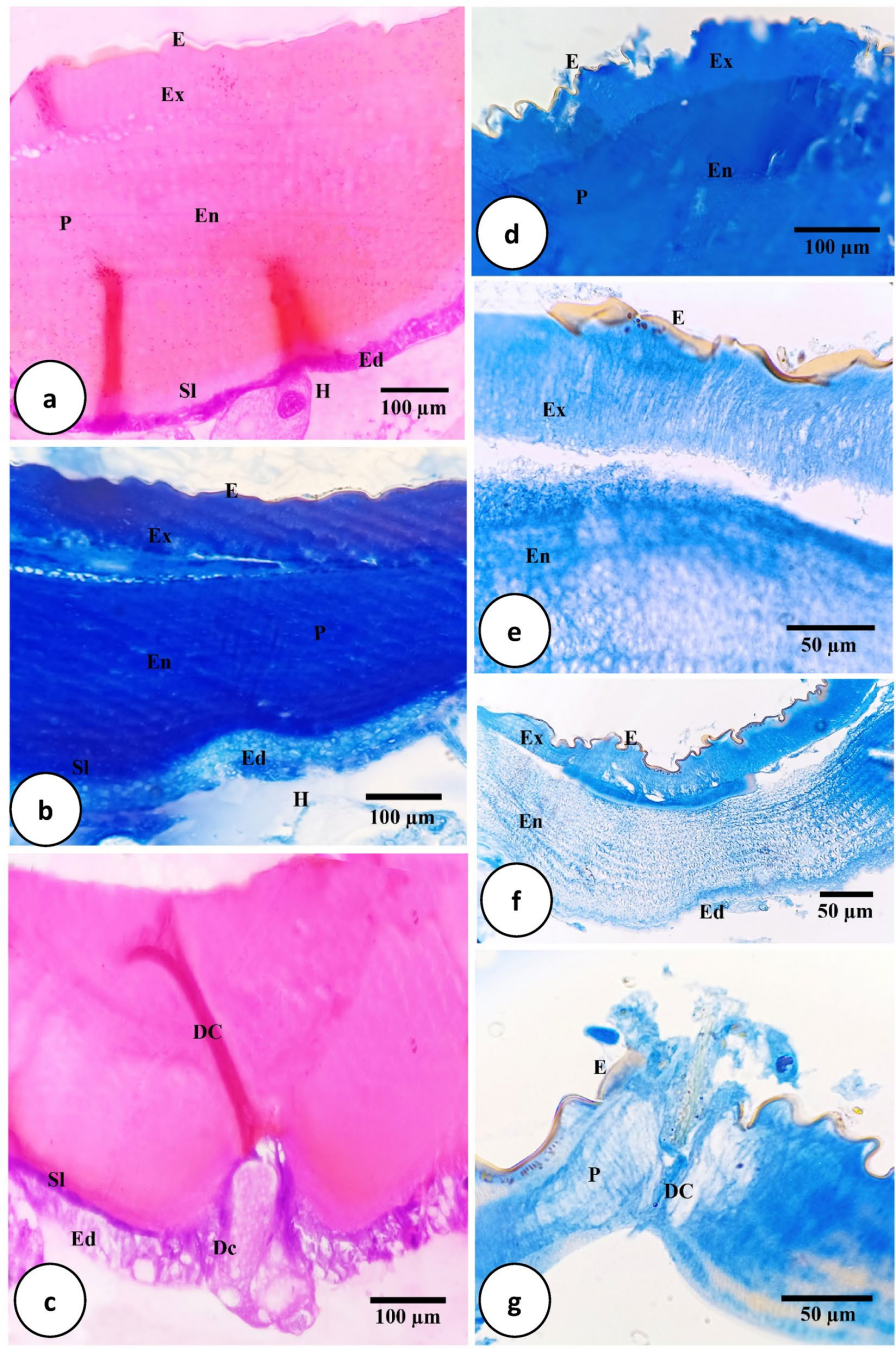
Light micrographs of transverse sections of *H. dromedarii* integument. (**a**) Paraffin section 4 days after feeding showing the heterogenous structure of the cuticle with an external epicuticle (E) and an internal procuticle (P) which differentiated into an outer exocuticle (Ex) and an inner endocuticle (En). Ed: Epidermis. Sl: Sub-cuticular layer. H: Heamocoal. HE stain. (**b**) Paraffin section 7 days after feeding showing the external epicuticle (E) and the internal procuticle (P) underlined with the sub-cuticular layer (Sl) and the epidermis (Ed). En: Endocuticle, Ex: Exocuticle. H: Heamocoel. MT stain. **c.** Paraffin section 4 days after feeding the integument with dermal gland that consists of 2–5 dermal cells (Dc) and dermal canal (DC). Ed: Epidermis; Sl: Sub-cuticular layer. HE stain. (**d**) Paraffin section 4 days after treatment showing erosion, damage or destruction in epicuticular layer (E) and abnormal appearance of all integument layers. En: Endocuticle, Ex: Exocuticle, P: Procuticle. MT stain. (**e**) Paraffin section 7 days after treatment showing abnormal appearance of cuticle layers, damage of the epicuticle (E), and separation between exocuticle (Ex) and endocuticle (En). MT stain. (**f**) Paraffin section 7 days after treatment separation between the exocuticle (Ex) and the endocuticle (En), and damaged epidermal cells (Ed). E: Epicuticle. MT stain. (**g**) Paraffin section 7 days after treatment showing complete degeneration of dermal gland cells as well as damage of dermal canal (DC). E: Epicuticle. P: Procuticle. MT stain.

**Fig. 4:**
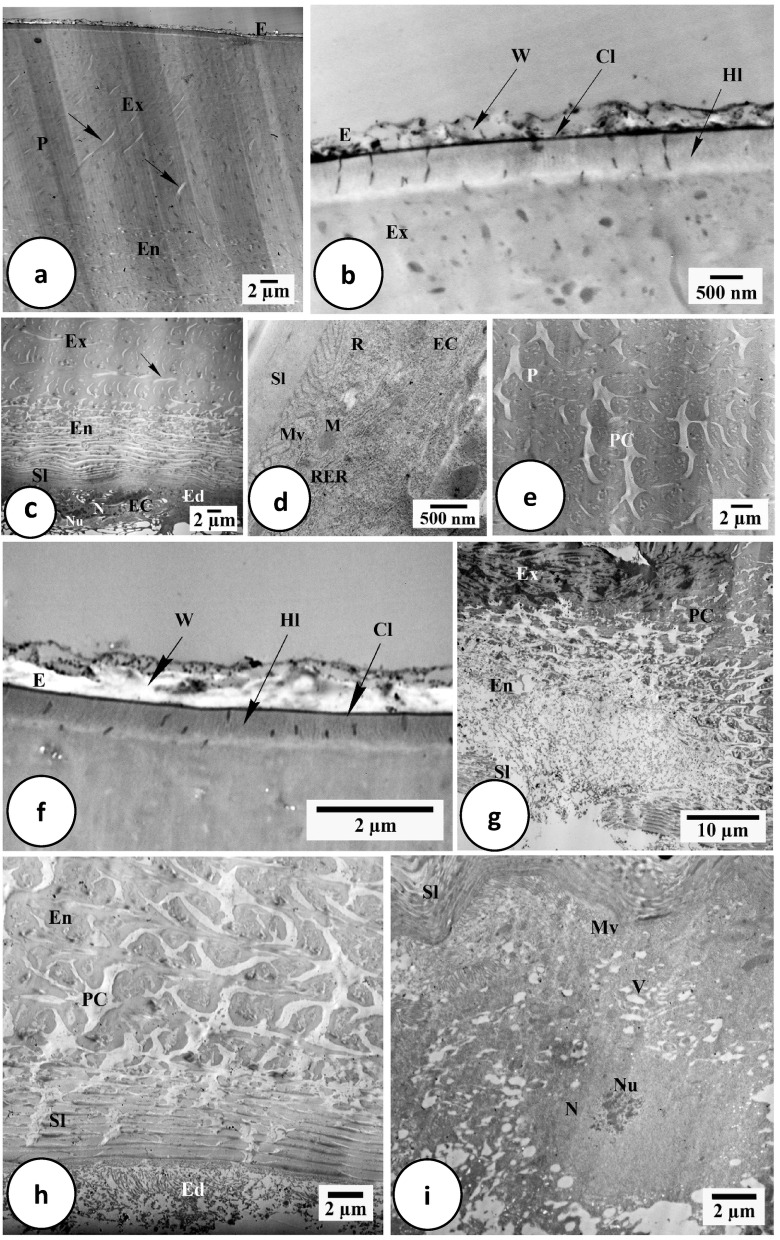
Transmission electron micrographs of *H. dromedarii* integument. (**a**) 4 days after feeding showing the outer highly electron dense epicuticle (E) and the inner procuticle (P) with the exocuticle (Ex), endocuticle (En) and pore canals (arrows). **(b**) 4 days after feeding the epicuticle (E) multilayered structure with the outer wax layer (W), the cuticulin layer (Cl) with high density, and less dense homogenous layer (Hl) which separates the epicuticle (E) and the exocuticle (Ex). (**c**) 7 days after feeding showing the procuticle (P) with outer exocuticle (Ex) and inner endocuticle (En), the sub-cuticular layer (Sl) and the epidermis layer (Ed) with epidermal cells (EC). Pore canals (arrow). N: Nucleus. Nu: Nucleolus. (**d**) 7 days after feeding showing the cytoplasm of epidermal cell (EC) containing free ribosomes (R), mitochondria (M) and numerous rough endoplasmic reticulum (RER). Plasma membrane of epidermal cell had numerous microvilli (Mv) that direct toward the sub-cuticular layer (Sl). (**e**) 4 days after feeding showing the spongy appearance of the procuticle (P) with the presence of pore canals (PC). (**f**) 7 days after treatment showing the epicuticle (E) layer with separation between its outer wax layer (W) and the cuticulin layer (Cl). Hl: Homogenous layer. (**g**) 7 days after treatment showing marked deterioration of cuticle layers. En: Endocuticle; Ex: Exocuticle; PC: Pore canals; Sl: Subcuticular layer. (**h**) 7 days after treatment showing loss of lamellar arrangement of the endocuticle (En), specious dilation of pore canals (PC), abnormal appearance of subcuticular layer (Sl) and damaged microvilli in epidermis (Ed). (**i**) 7 days after treatment showing the epidermal cell with highly vacuolated cytoplasm (V), irregularly shaped enlarged nucleus (N), fragmented nucleolus (Nu) and damaged microvilli (Mv). Sl: Subcuticular layer.

As revealed by light and transmission electron microscopical examination, the integument of *H. dromedarii* is divided into three main regions; the cuticle, subcuticular layer, and epidermis (Figs. 3a–c and 4a–e).

The exterior thin epicuticle and the interior thicker, more developed procuticle are the two main different layers that make up the cuticle’s heterogeneous appearance (Fig. 3a). After feeding, the cuticle’s thickness decreased from four to seven days (Fig. 3a,b).

The outer, thinner, and slightly folded layer is called the epicuticle (Fig. 3a,b). It responded faintly with HE stain (Fig. 3a) and stained orange or mild red with MT stain (Fig. 3b). On the other hand, it appeared as a multilayered, extremely electron-dense structure (Fig. 4a,b). Three distinct layers were observed in; wax, cuticulin, and a thick, less dense, homogenous layer (Fig. 4b). One characteristic of the cuticulin layer was its high density (Fig. 4b). A layer of wax was present above the cuticulin layer (Fig. 4b).

Four days of treatment, the epicuticle layer appeared damaged (Fig. 3d). In certain regions, it was either undetectable or appeared abnormal with additional folds (Fig. 3d). Additionally, the epicuticle layer, particularly the wax layer, seemed thicker after seven days of treatment compared to the untreated group (Fig. 3e). TEM examination revealed separation between wax and cuticulin layers (Fig. 4f).

The procuticle had two separate layers; the exocuticle, in contact with the epicuticle, and the endocuticle, near the epidermis (Figs. 3a, b, and 4a–c). Although it reacted less intensely with HE stain (Fig. 3a), the endocuticle’s staining affinity was lower than the exocuticle’s, allowing for the differentiation between two layers (Fig. 3a). Oppositely, the procuticle was stained dark blue using MT stain with undistinguishable layers (Fig. 3b). The chitin material in the exocuticle seemed randomly structured under an electron microscope, whereas the endocuticle showed layers of more electron-dense chitin material with electron-lucid matrix creating overlaying lamellae in between (Fig. 4c).

The treated integument’s procuticle layers (exocuticle and endocuticle) appeared thicker, disorganized, and their content extremely deteriorated (Fig. 3e). TEM observation revealed crumpled and disorganized lamellar arrangement found in the exocuticle and endocuticle (Fig. 4g).

The subcuticular layer appeared with a faint pink color when stained by HE stain (Fig. 3a); and a dark blue color when MT stain was used, compared to other layers (Fig. 3b). Under the electron microscope, it appeared as a dark dense area (Fig. 4c).

TEM examination of the treated ticks’ subcuticular layer showed an abnormal appearance and rupture in some areas (Fig. 4g). There seemed to be many vacuoles and damaged microvilli at the attachment site between the subcuticular layer and epidermis (Fig. 4h).

The epidermis is a single cell layer settled on a basal lamina separating the integument from the hemocoel (Fig. 3a). These cells appeared irregular under electron microscope, contained irregular nucleus with several heterochromatin patches and a visible nucleolus (Fig. 4c). Free ribosomes, mitochondria, and many rough endoplasmic reticulum cisternae were clearly observed (Fig. 4d). The plasma membrane of epidermal cells contained numerous microvilli directed toward the subcuticular layer and vesicles containing electron-dense material below them (Fig. 4d).

After extract treatment, the epidermal layer showed a significant disorganization and/or total destruction (Fig. 3f). After four and seven days of treatment, TEM showed abnormal features, including damaged microvilli, an irregularly shaped enlarged nucleus, a fragmented nucleolus, and degraded and severely vacuolated cytoplasm (Fig. 4i).

Throughout the epidermal layer, dermal glands were visible and connected to the exterior via a duct open on the cuticle surface (Fig. 3c). Each gland consisted of 2–5 large polygonal cells with faintly stained cytoplasm (Fig. 3c).

The treated ticks’ dermal glands showed enlarged and/or damaged dermal ducts with damaged cells (Fig. 3g).

The pore canals were seen to be a highly branching system of fine channels that penetrated the entire cuticle, connecting the epicuticle with the underlying epidermal cells (Fig. 4c and e). These canals were located in various directions inside the procuticle layer (Fig. 4c). They were anastomosing, ramified, and abundant in the endocuticle, while they were fewer and less ramified in the exocuticle (Fig. 4a and c). There was less electron-dense material inside canals, which were membrane-limited (Fig. 4e).

Following treatment, a fibrous-like structure was left behind by the endocuticle’s pore canals that exhibited a significant dilatation, particularly in the area connected to the subcuticular layer (Fig. 4h).

**Fig. 5:**
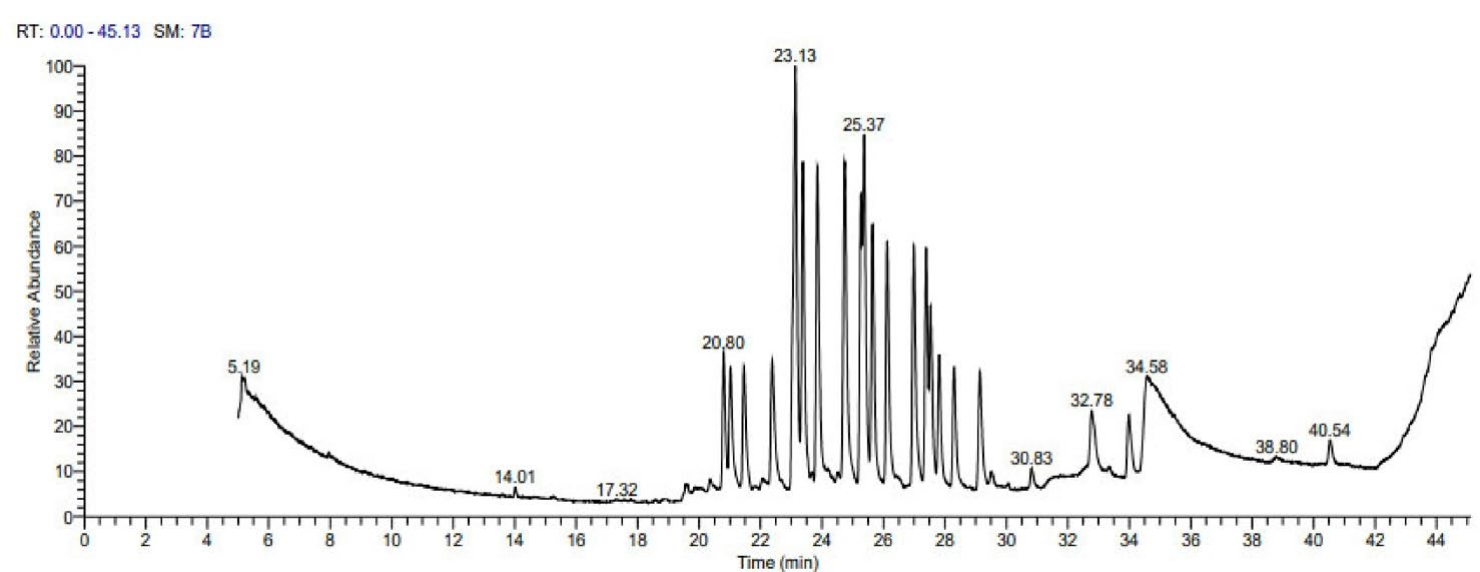
Volatiles profile of ethanolic extract of C*itrullus colocynthis* fruit by using GC–MS.

Using GC–MS, a wide variety of volatile components were present in the ethaonolic extract of *C. colocynthis*. Peak area, retention time, molecular weight, and molecular formula are all important factors in phytochemical compound identification. The identified volatiles are trichloromethane, 4-Oleoylmorpholine, cycloheptasiloxane, tetradecamethyl, hexacosane, spathulenol, decylbenzene, dodecylbenzene, undecylbenzene, eicosane, 1-phenyl, benzene, (1-butyloctyl), palmitic acid methyl ester, stearic acid, methyl linoleate, eicosadienoic acid, 9,12-octadecadienoic acid (Z,Z) and monostearyl maleate (Table 1 and Fig. 5).

**Table 1:** Volatiles content of ethanolic extract of *Citrullus colocynthis.*

Retention Time	Peak area %	Compound	Molecular formula	Molecular weight
5.12	1.06	Trichloromethane	CHCl3	118
14.02	0.19	4-Oleoylmorpholine	C22H41NO2	351
19.56	0.25	Cycloheptasiloxane, tetradecamethyl-	C14H42O7Si7	518
19.63	0.14	Hexacosane	C26H54	366
20.36	0.37	Spathulenol	C15H24O	220
20.80	2.98	Decylbenzene	C16H26	218
21.46	2.78	Dodecylbenzene	C18H30	246
23.13	11.19	Undecylbenzene	C17H28	232
23.85	7.18	Eicosane, 1-phenyl-	C26H46	358
25.37	7.86	Benzene, (1-butyloctyl)-	C18H30	246
27.39	5.56	Androstane	C19H32	260
28.29	2.71	Heneicosane, 11-phenyl-	C27H48	372
29.14	2.86	Benzene, (1-methyldodecyl)-	C19H32	260
29.52	0.33	Palmitic acid methyl ester	C17H34O2	270
30.83	0.45	Stearic acid	C18H36O2	284
32.78	1.81	Methyl linoleate	C19H34O2	294
33.98	1.65	Eicosadienoic acid	C20H36O2	308
34.55	1.78	9,12-Octadecadienoic acid (Z,Z)-	C18H32O2	280
40.54	0.57	Monostearyl maleate	C22H40O4	368

## Discussion

Ticks’ integument is an essential organ forming the exoskeleton, which envelops the entire body and serves as a physical barrier, support, and defense against external threats^48^. Furthermore, it controls the water balance and has the ability to quickly stretch and expand when female ticks engorge during the blood meal, which facilitates egg formation and tick reproduction^49^. For these reasons, evaluating the chemical effects on the integument seems to be a reliable and straight-forward strategy to estimate the entrance and possible action of chemicals in the internal organs of ticks^30,31^.

In the present SEM study, the dorsal integument of fed female *H. dromedarii* is greatly expanded with several folds. The same results were reported by Hackman^50^ in *Boophilus microplus*, Arruda et al.^51^ in *B. microplus*, Ghosh and Misra^52^ in *Amblyomma gervaisi*, Remedio et al.^53^ in *R. sanguineus* and Starck et al.^35^ in *Ixodes ricinus*. Numerous dermal gland pores and scattered setae were found in the dorsal integument during the current study. Estrada-Peña et al.^54^ reported nearly identical results for *I. inopinatus* and *I. ricinus*, Remedio et al.^53^ for *R. sanguineus*, and Patra et al.^55^ for *Aponomma varanense* using SEM.

According to the current findings using SEM examination of the fed *H. dromedarii* integument following immersion in 100 mg/mL *C. colocynthis* ethanolic extract Remedio et al.^30^ similarly observed the secretion on dermal gland openings in the *R. sanguineus* integument treated with neem plant extract. They suggested that it was caused due to disturbed cell metabolism of dermal glands. In the present examination, the thickness decreased in treated integument layers. This may be due to extract penetration, with failure of epidermal cells to replace it, as they were affected^32^.

Light (LM) and transmission electron microscopy (TEM) analysis of the fed female *H. dromedarii* integument in this work showed that the cuticle is differentiated into two distinct layers; a thin external epicuticle and a thick internal procuticle. This differentiation can be observed in the alloscutum of *H*. *asiaticum*^55^, *H*. *dromedarii*^57^, *I*. *ricinus*^58^, *H*. *analoticum*^59^, and *R*. *sanguineus*^53^. Similar to findings reported by Hackman and Filshie^60^ and Sonenshine and Roe^49^, LM in the present study of the cuticle revealed the presence of an outer, thinner, and slightly folded layer called the epicuticle. TEM revealed that it is extremely electron-dense and has a multilayered structure, including wax, cuticulin, and a less dense homogeneous layer. Similar findings were noted for *Haem. leporspalustris*^61^, *B. microplus*^62^, *H. asiaticum*^63^, *H. dromedrii*^64^, *H. anatolicum*^59^.

In the current study, the procuticle was made up of two separate sublayers; the exocuticle, which was in contact with the epicuticle, and the endocuticle, which was close to the epidermis. According to these results, these layers are easily observed and distinguishable in *B. microplus* and *B. decoloratus*^65^, *H. asiaticum*^56^, *H. dromedarii*^57^, *Amb. hebraeum*^66^, *R. appendiculatus*^67^, *B. microplus*^51^, *R. sanguineus*^53,68^, and *D. andersoni* and *D. variabilis*^49^.

According to results of the current histological study, the subcuticular layer was found below the endocuticle. Hackman^69^, Coons and Alberti^70^, and Remedio et al.^53^ identified this layer in *R. microplus*, *I. ricinus*, and *R. sanguineus*, respectively.

LM and TEM examination of the integument showed the presence of an epidermis that contained irregular shaped cells, each had an irregular nucleus with peripheral heterochromatin, dispersed euchromatin and a visible nucleolus, several free ribosomes, mitochondria, and rough endoplasmic reticulum. Additionally, microvilli with vesicles containing electron-dense material were observed oriented towards the sub-cuticular layer. Such observations were in great accordance with those previously mentioned^49,53,56,61,65,67,71,72^.

Throughout the epidermal layer, dermal glands were visible and connected to the outside through a duct that led to a cuticle surface aperture. Similarly, the present findings were detected in the integument of *R*. *microplus*^68^ and *H*. *asiaticum*^63^.

In the present study, LM examination of treated integument revealed damaged epicuticle with increased folds. TEM showed that the layers of wax and cuticulin were separated. The procuticle layer lacked lamellar structure and seemed less organized with coalesced layers. Additionally, there was a noticeable dilated pore canals in the endocuticle. The subcuticular layer has damaged microvilli and vacuoles, giving it an abnormal appearance. It was also noted that the epidermal cells and the subcuticular layer separated. The epidermal layer appeared with great disorganization, as its cells suffered from cytoplasmic degeneration and vacuolization, loss of cytoplasmic organelles, and damage of nuclear membrane and the nucleolus. Morphological alterations in epidermal cells indicate that the active ingredients of the extract were capable of crossing the cuticle barrier and reaching the epidermis^30^.

According to de Souza et al.^31^, changes in the epidermal cells of female *R. sanguineus* ticks treated with neem plant extract may result in long-term changes to the physiological characteristics of the epidermis and may even cause cell death due to the noticeable nuclear deformation^73^. The occurrence of nuclear alterations (characteristics of cell death) in cells of the epidermal layer can affect the synthesis and secretion of the cuticular components and hence leave the tick more susceptible to changes in the external environment^73^.

The presence of vacuoles in epidermal cells cytoplasm is one of morphological changes that occur when organisms are exposed to stress. This is an indication that the defense mechanism is activated, which would be taking place by isolation of damaged cytoplasmic components and organelles that have lost their function, then broken down by lysosomal enzymes^73–76^. This strategy allows cells to preserve their internal systems and thus the efficiency of their metabolism^77^. Cytoplasmic retraction of the epidermal cells or dilated intercellular gaps suggested changes in tight junctions and adhesion junctions, which are frequently found in lining cells^78^.

In the present investigation, the pore canals of treated females showed dilatation and disintegration, creating an empty network. The dermal glands were characterized by enlarged ducts with ruptured membranes, and damaged cells. The integumental glands’ enlargement suggests that the secretory cells are very active^79^, releasing lipids that serve as a protective barrier^67^.

Using adult immersion tests, several authors have reported similar effects of plant extracts on the integument of semiengorged and engorged females of various tick species, including *R. sanguineus*^30,31^, *Haem. longicornis*^16^, and *H. dromedarii*^33^. Similar results were found in *R. sanguineus* after using chemicals derived from plants^32^.

Gas chromatography-mass spectrometry (GC–MS) is the most widely used method for analyzing volatile chemicals^80–82^. It is a technique that combines the performance of gas chromatography, which allows the separation of compounds, and the performance of mass spectrometry, which enables the finding and identification of compounds according to their mass-to-charge ratio (m/z)^83^.

In this study, volatiles from *C. colocynthis* were found mainly in the plant extract’s fruit using GC-Mass^84^. Its seeds also rich in stearic and palmitic acids^85^ that exhibit biological activity against arthropods^86,87^, and insecticidal effects on *Melanaphis sacchari*^88^. According to Mohamed et al.^2^, GC–MS analysis of the *C. colocynthis* ethanolic extract showed a significant diversity of fatty acid contents, which are regarded as one type of volatiles. Among these components were linoleic acid, vaccenic acid, decanoic acid, carbamic acid, and oleic acid.

Volatile organic compounds (VOCs) are lipophilic with low molecular weights and high vapor pressures^83^. Their physical properties facilitate them to cross cell membranes^89^. Accordingly, it was suggested that all previous changes detected in the tick integument following treatment in the present study were attributed to the occurrence of volatiles such as methyl linoleate, octadecadienoic, palmitic and stearic acids. These compounds have lipophilic structures, and their penetration through the integument may disrupt cell membranes, altering their permeability and causing significant deformation in all integument layers. This suggestion is in great agreement with some authors^90–92^.

## Conclusion

Histological examinations of fed female *Hyalomma dromedarii* integument after *Citrullus colocynthis* extract treatment revealed symptoms of great damage to all cuticle layers and the epidermis. Alterations in the morphology of epidermal cells because of the aggression of plant components on the cuticle were accompanied by changes in their physiological status. The data provided suggest that the used plant extract may be applied for biological control of *H. dromedarii*.

## Data Availability

We are the authors assure that all data and materials support the published claims and comply with field standards. The data are mentioned in the manuscript and will be available after publication.
